# Hepatitis B virus stimulates G6PD expression through HBx-mediated Nrf2 activation

**DOI:** 10.1038/cddis.2015.322

**Published:** 2015-11-19

**Authors:** B Liu, M Fang, Z He, D Cui, S Jia, X Lin, X Xu, T Zhou, W Liu

**Affiliations:** 1 Department of Biochemistry and Molecular Biology, Program in Molecular Cell Biology, Zhejiang University School of Medicine, Hangzhou, Zhejiang 310058, China; 2 Department of Surgery, First Affiliated Hospital, Zhejiang University School of Medicine, Hangzhou, Zhejiang 310003, China; 3 Collaborative Innovation Center for Diagnosis and Treatment of Infectious Disease, First Affiliated Hospital, Zhejiang University School of Medicine, Hangzhou, Zhejiang 310003, China

## Abstract

Metabolic reprogramming is a hallmark of physiological changes in cancer. Cancer cells primarily apply glycolysis for cell metabolism, which enables the cells to use glycolytic intermediates for macromolecular biosynthesis in order to meet the needs of cell proliferation. Here, we show that glucose-6-phosphate dehydrogenase (G6PD), the first and rate-limiting enzyme of the pentose phosphate pathway, is highly expressed in chronic hepatitis B virus (HBV)-infected human liver and HBV-associated liver cancer, together with an elevated activity of the transcription factor Nrf2. In hepatocytes, HBV stimulates by its X protein (HBx) the expression of G6PD in an Nrf2 activation-dependent pathway. HBx associates with the UBA and PB1 domains of the adaptor protein p62 and augments the interaction between p62 and the Nrf2 repressor Keap1 to form HBx–p62–Keap1 complex in the cytoplasm. The aggregation of HBx–p62–Keap1 complexes hijacks Keap1 from Nrf2 leading to the activation of Nrf2 and consequently G6PD transcription. Our data suggest that HBV upregulates G6PD expression by HBx-mediated activation of Nrf2. This implies a potential effect of HBV on the reprogramming of the glucose metabolism in hepatocytes, which may be of importance in the development of HBV-associated hepatocarcinoma.

Cancer is a disease with complex metabolic perturbations. Unlike normal differentiated cells that rely mainly on oxidative phosphorylation for energy production, cancer cells uptake large quantities of glucose and adopt primarily glycolysis for ATP generation even in the presence of ample oxygen.^1^ This metabolic characteristic promotes in cancer cells the glycolysis-associated biosynthetic processes including the pentose phosphate pathway (PPP), enabling cancer cells to utilize glucose for the biosynthesis of macromolecules to support their rapid division.^2^ The PPP provides cells with ribose 5-phosphate required for *de novo* nucleotide biosynthesis, and with the reduced form of nicotinamide adenine dinucleotide phosphate (NADPH) for reductive biosynthesis such as the production of lipid.

Glucose-6-phosphate dehydrogenase (G6PD) is the first and rate-limiting enzyme in the PPP. G6PD converts glucose-6-phosphate into 6-phosphogluconolactone with a concomitant production of NADPH. Elevated G6PD expression and activity have been observed in breast, gastric, and prostatic cancers.^3, 4, 5^ When enhanced G6PD upregulates apoptosis-inhibitory factor Bcl-2 and Bcl-xl, and the cell cycle-related proteins,^6^ ectopic expression of G6PD promotes cell growth and the development of tumor in nude mice.^7^ It has been shown that the tumor suppressor p53 binds to G6PD and inhibits G6PD activity while many p53 mutants lost the G6PD-inhibitory activity.^8^ Promotion of cancer cell proliferation by TAp73, a p53-related protein, is also attributed to an upregulated G6PD.^9^ These data suggest that in addition to contribute to cancer growth and survival, *G6PD* also serves as an oncogene.

NF-E2-related factor 2 (Nrf2) is a master transcriptional factor responsible for the regulation of a number of antioxidant and cytoprotective genes, primarily in response to electrophiles and reactive oxygen species (ROS).^10^ Under normal conditions, Nrf2 is constantly associated with its inhibitor Kelch-like ECH-associated protein 1 (Keap1) and degraded by the proteasomes. Elevated intracellular ROS and accumulation in electrophiles lead to oxidation of key cysteine residues on Keap1 disrupting Keap1–Nrf2 interaction. Nrf2 then shifts into the nucleus and activates the transcription of cytoprotective genes that encode detoxifying enzymes. Recently, accumulating evidence has demonstrated a constitutive stabilization of Nrf2 in various human cancers;^11, 12, 13, 14^ and cancers with high Nrf2 level are associated with poor prognosis.^10, 11^ In addition, elevated Nrf2 activity enhances the expression of PPP enzymes including G6PD, and accelerates cancer cell proliferation.^15^ Deletion of Nrf2 can reduce carcinogen-induced lung tumor development in mice,^16^ and the oncogenes *K-Ras* and *Myc* specifically target the expression of Nrf2 in cancer cells.^17^ These data suggest that Nrf2 is an important mediator of oncogenesis. Intriguingly, it has recently been shown that accumulation of p62, an autophagy-adaptor protein, can cause a persistent activation of Nrf2 contributing to the growth of human hepatocellular carcinoma (HCC).^14, 18^

In this study, using clinical specimen and cultured cells, we have investigated the potential influence of hepatitis B virus (HBV), a major pathogenic factor for HCC worldwide, on the metabolism of hepatocytes by focusing on the expression of G6PD. We found that HBV upregulates G6PD in hepatocytes which relies on its X protein (HBx)-mediated activation of Nrf2. HBx interacts with p62 and Keap1 to generate HBx–p62–Keap1 aggregates in the cytoplasm leading to the nuclear translocation and activation of Nrf2.

## Results

### HBV upregulates G6PD expression

**Figure 1:**
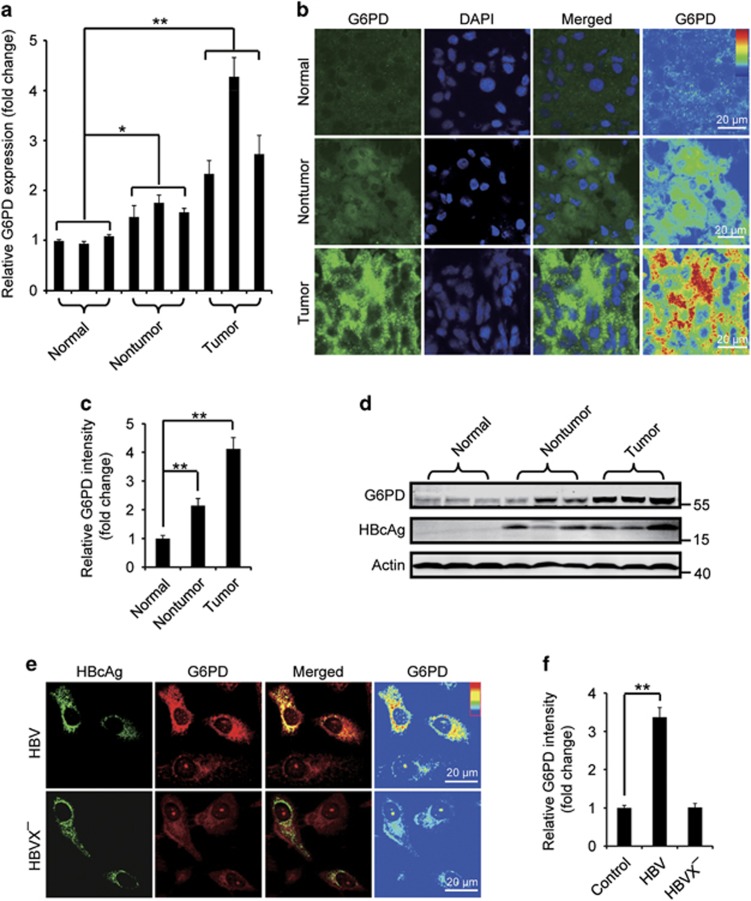
HBV upregulates G6PD expression through HBx protein. (**a**) The relative *G6PD* mRNA levels in normal liver tissues, liver tumor or non-tumor tissues. The data are presented as mean±S.E.M. of triplicate experiments. (**b**) Representative immunostaining of G6PD in the human tissues. (**c**) Quantification of the relative mean G6PD intensity from 10 randomly selected visual fields in each human tissue. (**d**) Western blot analysis of G6PD in the human tissues. (**e**) Huh7 cells were transfected with the HBV genomic DNA (HBV) or the HBx-negative HBV genomic DNA (HBVX^−^). At 48 h after transfection, the cells were stained with HBcAg and G6PD antibodies. (**f**) Quantification represents the relative fluorescence intensity of G6PD in cells with or without expression of HBV or HBVX^−^, *n*=30. All the quantitative data are presented as mean±S.E.M., **P<0.01, *P<0.05. Scale bars, 20 *μ*m

To test whether HBV infection may potentially modulate the PPP in hepatocellular metabolism, we began with the investigation of the expression of G6PD, the rate-limiting enzyme of the PPP, in liver tumor and non-tumor tissues from subjects with HBV-associated HCC, and in samples from normal control individuals. We found that the *G6PD* mRNA level in the tumor (10/13=76.9%) and non-tumor tissues (7/13=53.8%) dramatically increased compared with the normal group (Figure 1a and Supplementary Figure 1a). Immunostaining and western blot affirmed an elevation of G6PD protein level in the same samples while the tumor samples showed a higher G6PD than that in the non-tumor samples (Figures 1b–d). In search of evidence that HBV infection stimulates G6PD expression, we checked G6PD protein in human hepatoma Huh7 cells expressing HBV genomic DNA, and HepG2.2.15 cell, a HBV-replicating cell line. We found in the cells that HBV DNA expression dramatically raised G6PD protein level (Figures 1e and f and Supplementary Figure 1b). Interestingly, expression of HBVX^−^ DNA, an HBV genomic DNA that is incapable of expressing HBx protein, failed to increase G6PD level, suggesting a critical role of HBx in the stimulation (Figures 1e and f).

**Figure 2:**
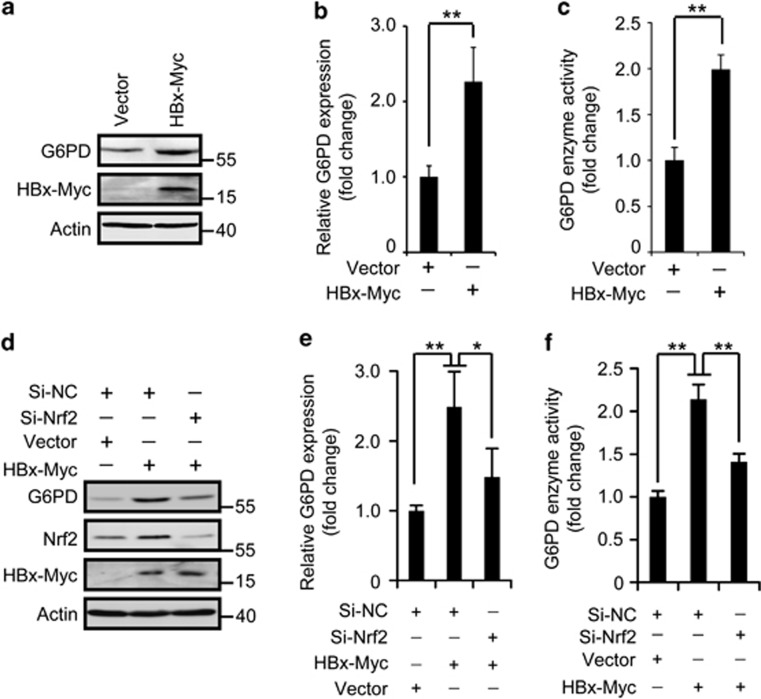
HBx enhances G6PD activity via Nrf2. (**a** and **b**) Western blot (**a**) and qRT-PCR (**b**) analysis of G6PD expression in Huh7 cells expressing HBx-Myc. (**c**) The relative G6PD enzyme activity in cells treated as in (**a**). (**d**–**f**) The protein level (**d**), mRNA level (**e**), and enzyme activity (**f**) of G6PD in Huh7 cell with or without HBx-Myc expression and Nrf2 RNAi. All the statistical data are presented as mean±S.E.M. of triplicate experiments. *P<0.05; **P<0.01

To verify the role of HBx in mediating HBV's effect on G6PD expression, hepatitis B surface antigen (HBsAg), hepatitis B core antigen (HBcAg) or HBx, was transfected respectively in human hepatic L02 cells and Huh7 cells. Significantly, HBx, but not HBsAg or HBcAg, hiked G6PD at both mRNA and protein levels in L02 and Huh7 cells (Figures 2a and b and Supplementary Figures 1c and d). Considering the higher transfection efficiency, Huh7 cells were used in subsequent investigation. Using glucose-6-phosphate as a substrate, enzyme activity analysis demonstrated a boosted total G6PD activity in HBx-Myc-transfected cells (Figure 2c). Taken together, these results suggest that HBV upregulates G6PD in hepatocytes through HBx.

### HBV stimulates Nrf2 activation

**Figure 3:**
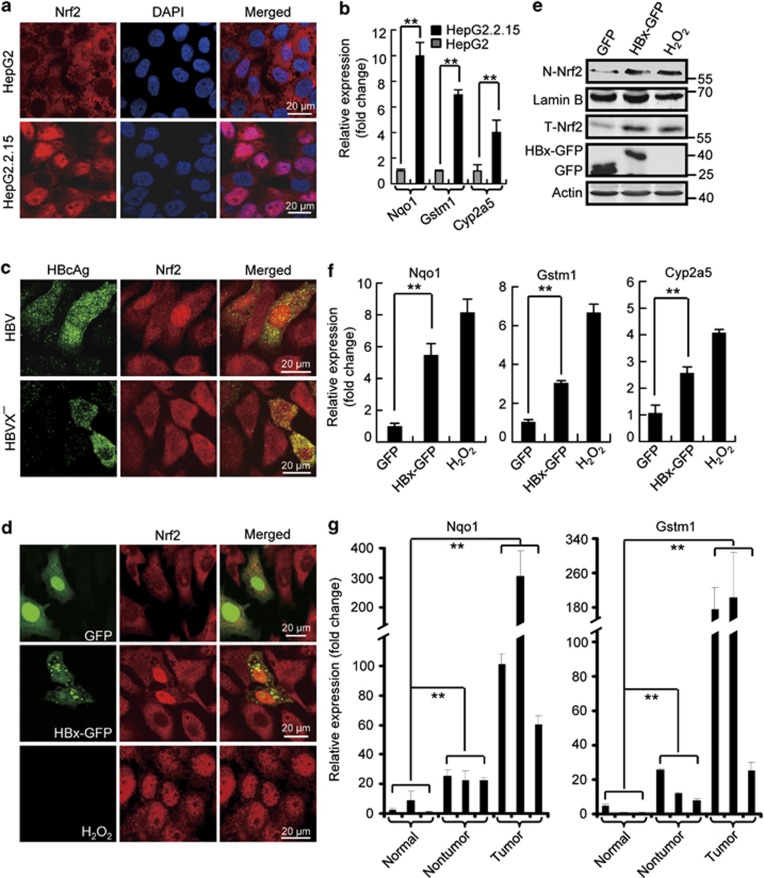
HBV stimulates Nrf2 activation. (**a**) Immunostaining of Nrf2 in HepG2 and HepG2.2.15 cells. Note the nuclear distribution of Nrf2 in HepG2.2.15 cells. (**b**) The mRNA levels of *Nqo1*, *GSTm1*, and *Cyp2a5* measured by qRT-PCR in HepG2 and HepG2.2.15 cells. (**c**) Huh7 cells transfected with HBV genomic DNA (HBV) or HBx-negative HBV genomic DNA (HBVX^−^) were stained with HBcAg and Nrf2 antibodies at 48 h after transfection. (**d**) Huh7 cells were either treated with 250 *μ*M H_2_O_2_ for 4 h or transfected with GFP or HBx-GFP for 48 h. Then, the cells were fixed and stained with Nrf2 antibody. (**e**) Western blot analysis of Nrf2 protein in nuclear extracts (N-Nrf2) and cell lysis (T-Nrf2) in cells treated as in (**d**). Lamin B was used as an internal control of nuclear fraction. (**f**) The mRNA levels of *Nqo1*, *GSTm1*, and *Cyp2a5* measured by qRT-PCR in cells treated as in (**d**). (**g**) *Nqo1* and *GSTm1* mRNA levels in normal liver tissues and HBV-infected non-tumor or tumor tissues. All the statistical data are presented as mean±S.E.M. of triplicate experiments. **P<0.01. Scale bars, 20 *μ*m

Nrf2 contributes to cancer development by targeting not only the typical cytoprotective genes but also genes involved in cell metabolism including G6PD.^15^ To test a potential role of Nrf2 in HBV-triggered upregulation of G6PD, we performed Nrf2 RNA interference and checked G6PD expression and activity in Huh7 cells. Knockdown of Nrf2 evidently decreased the mRNA and protein levels and total activities of G6PD induced by HBx-Myc expression (Figures 2d–f), indicating wherein an essential role of Nrf2. We then investigated the effect of HBV/HBx on Nrf2 activity. First, we looked over the intracellular distribution of Nrf2. In control HepG2 cells, Nrf2 is primarily localized to the cytoplasm. But in HepG2.2.15 cells Nrf2 showed a typical nuclear distribution (Figure 3a), indicating an activation of Nrf2. We also measured the expression of known Nrf2 target genes including NAD(P)H quinone oxidoreductase 1 (*Nqo1*), glutathione S-transferase M1 (*Gstm1*), and cytochrome P450 2A5 (*Cyp2a5*) in HepG2 and HepG2.2.15 cells. Apparently, HepG2.2.15 cells possessed higher level of each of the genes compared with that in HepG2 cells (Figure 3b). Finally, we transfected in the cells the HBV DNA and HBVX^−^ DNA and checked Nrf2 distribution. When Nrf2 showed a typical cytoplasmic distribution in non-transfected cells or cells expressing HBVX^−^ DNA, Nrf2 in HBV DNA-expressing cells was localized primarily to the nucleus (Figure 3c), indicating an HBx-mediated Nrf2 activation. This action of HBx was confirmed in cells expressing GFP-tagged HBx (HBx-GFP). Similar to that in cells treated by H_2_O_2_, a classic Nrf2 activator, expression of HBx, but not HBsAg or HBcAg, shifted cytoplasmic Nrf2 to the nucleus in L02 and Huh7 cells (Figure 3d and Supplementary Figure 2). The nuclear translocation of Nrf2 was further proved by a subcellular fractionation showing increased Nrf2 in the nucleus of HBx-expressing Huh7 cells (Figure 3e). Consistently, the expression of Nrf2 target genes, *Nqo1*, *Gstm1*, and *Cyp2a5*, was enhanced by HBx-GFP expression (Figure 3f).

Nrf2 activity was also examined in liver tissues from subjects with or without HBV-associated HCC by measuring the expression of Nrf2 target genes. Compared with the normal samples from healthy individuals, the non-tumor and tumor samples from HCC patients showed increased expression of the genes representing augmented Nrf2 activity (Figure 3g).

Taken together, these results suggest that HBV stimulates Nrf2 activation in hepatocytes.

### HBx stimulates p62–Keap1 interaction

**Figure 4:**
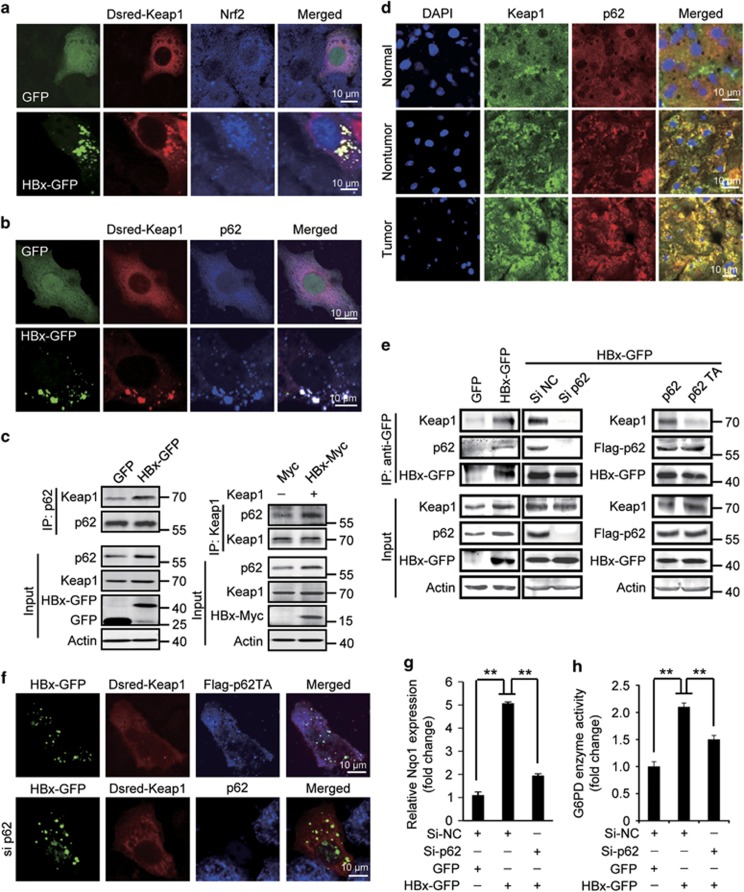
HBx-stimulated p62–Keap1 interaction is required for HBx-induced Nrf2 activation. (**a**) Localization of Dsred-Keap1 and Nrf2 in HBx-GFP-transfected Huh7 cells. (**b**) Colocalization of Dsred-Keap1 and p62 in HBx-GFP-expressing Huh7 cells. (**c**) Co-immunoprecipitation of Keap1 with p62 (left panel) or p62 with Keap1 (right panel) in HBx-expressing cells. (**d**) Immunostaining of p62 and Keap1 in normal liver tissues and HBV-infected non-tumor or tumor tissues. (**e**) Co-immunoprecipitation of HBx-GFP, p62, and Keap1 in HBx-GFP-expressing cells transfected without or with p62 siRNAs, Flag-p62, or Flag-p62T352A (p62TA). (**f**) Localization of Dsred-Keap1 in HBx-GFP-transfected cells with Flag-p62TA expression (upper panel) or p62 RNAi (lower panel). (**g**) *Nqo1* mRNA level measured by qRT-PCR in HBx-GFP-expressing cells with or without p62 RNAi. (**h**) G6PD enzyme activity in cells treated as in (**g**). All the statistical data are presented as mean±S.E.M. of triplicate experiments. **P<0.01. Scale bars, 10 *μ*m

Because the Nrf2 activity is mainly regulated by its interaction with Keap1, to investigate the mechanism by which HBx stimulates Nrf2 activation, we began with the observation of intracellular Keap1 localization. In Huh7 cells, expression of HBx-GFP but not GFP as a control, when endogenous cytoplasmic Nrf2 shifted to the nucleus, in the same cells, coexpressed Dsred-Keap1 formed in the cytoplasm aggregates with HBx-GFP (Figure 4a), implying an interaction between Keap1 and HBx. We then tested the potential role of p62 in mediating the Keap1–HBx interaction, because HBx can cause in hepatocytes the accumulation of p62,^19^ and elevated p62 results in Keap1 aggregation and Nrf2 activation through interaction with Keap1.^20^ We found a colocalization of p62 to Keap1–HBx aggregates in HBx-GFP and Dsred-Keap1-coexpressing cells (Figure 4b). In addition, in HBx-expressing cells, co-immunoprecipitation of p62 and Keap1 dramatically increased, indicating an enhanced interaction of the two proteins (Figure 4c). Promoted p62–Keap1 interaction was also observed in HepG2.2.15 cells compared with HepG2 cell (Supplementary Figure 3a). Furthermore, in liver tissue samples, in contrast to the normal liver tissue in which Keap1 and p62 were dispersed in the cytoplasm, the tumor and non-tumor samples from HBV-associated HCC patients showed a palpable colocalization of p62 and Keap1 forming in the cytoplasmic the p62–Keap1 aggregates, with more such aggregates in the tumor tissues (Figure 4d). These data suggested a stimulating effect of HBV on p62–Keap1 interaction through HBx. We further examined the HBx–Keap1 interaction in p62-deficient cells. In Huh7 cells expressing HBx-GFP, immunoprecipitation of HBx using GFP antibody resulted in a co-precipitation of endogenous Keap1 and p62 (Figure 4e). However, HBx-GFP failed to co-precipitate Keap1 in p62 knockdown cells or in cells overexpressing p62T352A, a p62 mutant defective in Keap1 interaction,^20^ while a strong co-precipitation of p62T352A with HBx was still detectable (Figure 4e). In addition, in p62 knockdown cells or cells expressing Flag-p62T352A, Keap1 could no more be recruited to the HBx punctual aggregates (Figure 4f). These data suggest that HBx can drive in hepatocytes the formation of HBx–p62–Keap1 complexes in which the recruitment of Keap1 relies on its interaction with p62.

At last, we examined the role of HBx–p62–Keap1 complex formation in the HBV/HBx-stimulated Nrf2 activation, by knocking down p62 in HepG2.2.15 cells and HBx-expressing Huh7 cells. Significantly, knockdown of p62 decreased *Nqo1* expression in both cells (Figure 4g and Supplementary Figure 3b). The activity of G6PD was also attenuated by p62 RNAi in Huh7 cells expressing GFP-HBx (Figure 4h). These results therefore suggest a crucial role of the formation of HBx–p62–Keap1 complexes in HBx-triggered Nrf2 activation and G6PD expression.

### Immobilization of Keap1 in the HBx–p62–Keap1 aggregates

**Figure 5:**
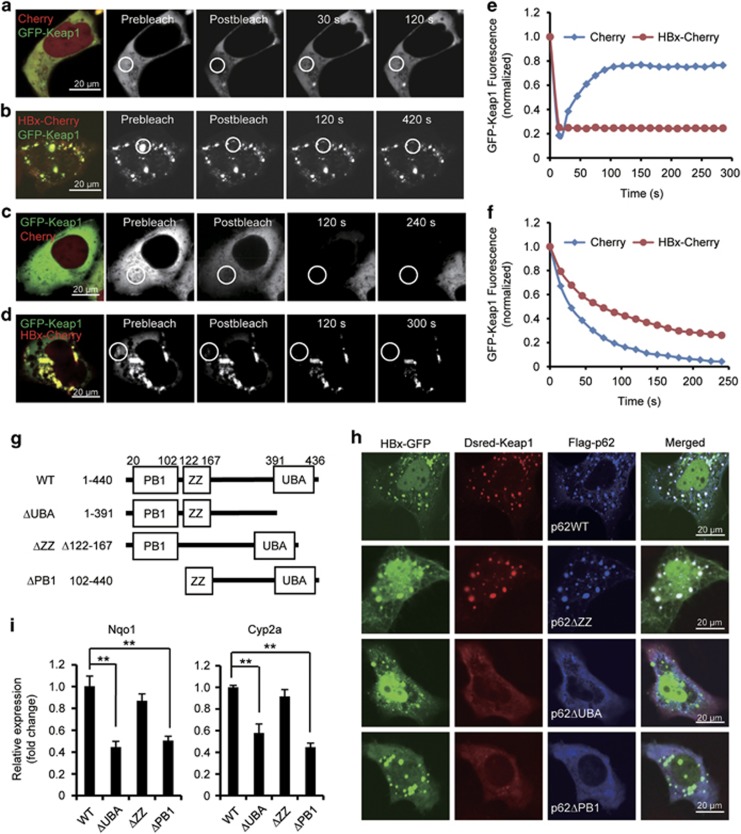
HBx immobilizes Keap1 in HBx–p62–Keap1 aggregates. (**a** and **b**) Huh7 cells expressing Cherry and GFP-Keap1 (**a**) or HBx-Cherry and GFP-Keap1 (**b**) were imaged before and after photobleaching the indicated region (white circles) by high intensity 488 nm laser light. Note the speed of fluorescence recovery in the photobleached region. (**c** and **d**) Repeated photobleaching of a cytoplasmic region (white circles) led all GFP-Keap1 fluorescence to be lost within a cell expressing GFP-Keap1 and Cherry (**c**), but not from the GFP-Keap1-HBx-Cherry aggregates in a cell expressing GFP-Keap1 and HBx-Cherry (**d**). (**e**) Quantification of the GFP-Keap1 signals in (**a**) and (**b**). (**f**) Quantification of the GFP-Keap1 signals in (**c**) and (**d**). (**g**) Schematic representation of the structure of p62 and the truncated mutants. (**h**) Localization of Dsred-Keap1 in cells expressing HBx-GFP with Flag-p62 or each of the Flag-tagged truncated p62 mutants. (**i**) Relative mRNA levels of *Nqo1* and *Cyp2a5* measured by qRT-PCR in cells transfected with HBx and p62 or HBx and each of the truncated p62 mutants. Data are presented as mean±S.E.M. of triplicate experiments. **P<0.01. Scale bars, 20 *μ*m

Given that HBx interacts with p62 and Keap1 to form HBx–p62–Keap1 aggregates in the cytoplasm, and the interactions contribute to the HBV-stimulated Nrf2 activation, a possible mechanism is that Keap1 is stuck in the aggregates leading to an abated inhibition of Nrf2. To address this, we checked whether Keap1 is associated with the aggregates in a dynamic on/off or in/out manner. First, we carried out fluorescence recovery after photobleaching (FRAP) in living cells expressing GFP-Keap1. A cytoplasmic region of GFP-Keap1 was photobleached and recovery of the fluorescence was monitored over time. Upon photobleaching, we observed a rapid recovery from the non-bleached pool, with the original prebleached fraction of fluorescence clearly restored in less than 100 s (Figures 5a and e). In contrast, in GFP-Keap1 and HBx-Cherry cotransfected cells, the fluorescence recovery of GFP-Keap1 aggregated with HBx-Cherry was dramatically slowed, with a great proportion of the initial GFP-Keap1 fluorescence failing to recover, even in a much longer time after photobleaching (Figures 5b and e), suggesting that this proportion of Keap1 was irreversibly bound to the aggregates. The irreversible binding of Keap1 to the aggregated pool was further confirmed by fluorescence loss in photobleaching (FLIP). GFP-Keap1 fluorescence in a small cytoplasmic region of the cell expressing GFP-Keap1 and Cherry was bleached repetitively. After about 200 s, the fluorescence signals were completely lost in the areas outside the region, indicating rapid diffusion between the bleached and unbleached areas (Figures 5c and f). However, in cells expressing GFP-Keap1 and HBx-Cherry, repetitive photobleaching of a similar cytoplasmic pool failed to cause the loss of GFP-Keap1 from the GFP-Keap1 and HBx-Cherry puncta, indicating a fixation of GFP-Keap1 in the aggregates (Figures 5d and f). These results suggest that the HBx–p62–Keap1 complexes are able to immobilize Keap1 in the formed aggregates.

To further investigate whether fix of Keap1 in the HBx–p62–Keap1 aggregates contributes to HBx-triggered Nrf2 activation, we created different truncated p62 mutants (Figure 5g), and the HBx–p62–Keap1 aggregate formation and Nrf2 activation were analyzed. We found that when coexpressed in cells, HBx and p62ΔZZ, a mutant p62 lacking the ZZ-type zinc finger domain, and Keap1, can still interact with each other to form the aggregates (Figure 5h and Supplementary Figure 4). However, in cells coexpressing HBx with p62ΔUBA or p62ΔPB1, two p62 mutants lacking the ubiquitin-associated domain or the Phox and Bem1p domain that fail to aggregate,^21^ HBx was not able to interact with Keap1 (Supplementary Figure 4), and they failed to form in the cytoplasm the aggregates (Figure 5h). Correspondingly, expression of p62ΔUBA or p62ΔPB1, but not p62ΔZZ, disrupted HBx-stimulated expression of Nrf2-targeted genes *Nqo1* and *Cyp2a5* (Figure 5i). These data suggest that by immobilization of Keap1, the formation of HBx–p62–Keap1 complex initiated by HBx is essential to HBx-stimulated Nrf2 activation.

### Nrf2 and G6PD are required for HBx-promoted cell proliferation

**Figure 6:**
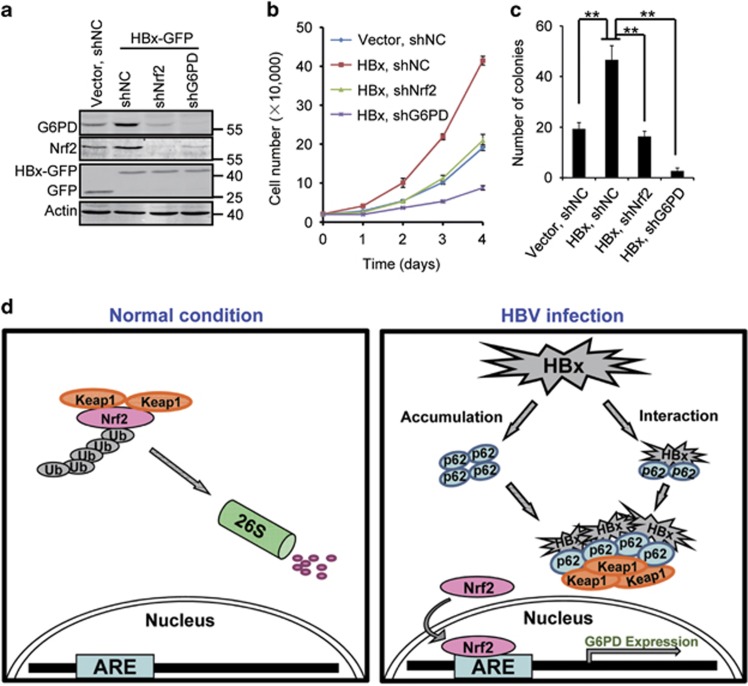
Nrf2 and G6PD are required to HBx-promoted cell proliferation. (**a**) G6PD and Nrf2 protein levels in HBx-GFP cells with the expression of indicated shRNAs. (**b**) Cell proliferation assay of the cells in (**a**). Data are presented as mean±S.E.M. of triplicate experiments. (**c**) Colony formation assay of the cells in (**a**). Colonies in three randomly chosen fields per well were quantified. Data are presented as mean±S.E.M. of triplicate experiments. **P<0.01. (**d**) Schematic model for HBV-stimulated Nrf2 activation and G6PD expression. In HBV-infected hepatocytes, HBx on the one hand results in the accumulation of p62 through inhibition of autophagic flux, and on the other hand interacts with Keap1 through p62, enabling the formation of HBx–p62–Keap1 aggregates in the cytoplasm. The aggregates hijack Keap1 from Nrf2 leading to Nrf2 activation and G6PD expression

Both Nrf2 and G6PD promote tumorigenesis.^7, 14^ To investigate a potential involvement of HBx-stimulated Nrf2 activation and G6PD expression in HBV-associated hepatocarcinogenesis, we checked the proliferation of HepG2 cells stably expressing HBx-GFP, with or without Nrf2 or G6PD knockdown. Consistent with previous reports,^22, 23, 24^ HBx-GFP expression enhanced proliferation of HepG2 cells (Figure 6b), and knockdown of Nrf2 or G6PD significantly inhibited HBx-GFP-stimulated cell proliferation (Figures 6a and b). We also performed colony formation assay to dissect the function of Nrf2 and G6PD. We found that knockdown of either Nrf2 or G6PD significantly repressed HBx-stimulated colony formation of HepG2 cells in soft agar (Figure 6c and Supplementary Figure 5).

## Discussion

Chronic HBV infection is worldwide one of the major causes for primary liver cancer, and the molecular mechanism for HBV-associated HCC remains elusive. Here, we demonstrate that HBV enhances in hepatocytes the expression of G6PD, the rate-limiting enzyme of the PPP, by activating Nrf2. Formation of HBx–p62–Keap1 aggregates in the cells that hijack Keap1 from Nrf2 underlies the mechanisms of HBV-stimulated Nrf2 activation for G6PD expression.

HBx has been suggested as a major pathogenic protein for HBV-associated HCC. However, previous studies have mostly focused on the action of HBx in the intracellular signal transduction pathways. HBx-mediated upregulation of G6PD and potential reprogramming of hepatocyte metabolism may bring new insights into the field. Viruses rely primarily on the metabolic capabilities of host cell to provide macromolecular precursors and energy to fuel their replication.^25, 26^ It is known that Hepatitis C virus and human cytomegalovirus are capable of enhancing nucleotide and lipid biosynthesis through upregulating glycolysis in their host cells.^27, 28^ Enhancement of the PPP by HBx-mediated upregulation of G6PD may explain on one hand that metabolic perturbations such as the use of nucleoside analogs can suppress HBV production,^29, 30^ and on the other hand the essential role of HBx in HBV replication.^31^ It is reasonable to speculate that through elevation of G6PD, HBV promotes PPP enabling host cell to utilize glucose for the biosynthesis of macromolecules to support their expensive proliferation which may be closely related to the development of HBV-associated HCC.

Notably, this is the first study showing G6PD overexpression in HBV-associated HCC, which correlates well with the results demonstrating HBx upregulates G6PD in cultured hepatocytes. As a pivotal initiator of the PPP, G6PD is highly regulated at the level of transcription, and in many cases by the transcription factor SREBP.^32^ Here, we have identified Nrf2 as a regulator of G6PD expression stimulated by HBx. This conclusion is also supported by the study showing that Nrf2 activation redirects glucose into anabolic pathways by transcriptional activation of PPP genes including G6PD.^15^ Nevertheless, our data do not exclude a potential involvement of other transcriptional regulators such as hypoxia-inducible factor 1 (HIF-1) in HBx-stimulated G6PD expression, because it has recently been demonstrated that activation of HIF-1 can induce G6PD expression,^33^ and HBx is able to activate HIF-1.^34^

Considering that Nrf2 serves as an intracellular redox sensor and a master transcriptional regulator for a great many antioxidants, and G6PD-mediated PPP pathway is responsible for NADPH production, HBV-stimulated Nrf2 activation might also be a result of elevated intracellular ROS, because HBx is able to increase ROS levels.^24, 35^ Nevertheless, we revealed that the activation of Nrf2 by HBx is mediated by an alternative more direct way. Clearly, we show that through interacting with the adaptor protein p62, HBx results in the formation of HBx–p62–Keap1 complexes, which further accumulates to generate the aggregates of the proteins in the cytoplasm. Identification of the interaction domain in p62 and application of the p62 mutant that failed to interact with Keap1 allowed us to determine that the formation of the complexes is initiated by HBx, and p62 has a pivotal role in bridging HBx and Keap1. Further, real-time imaging and photobleaching techniques ensured the investigation of the mobility of these proteins in living cells. Our data indicate that the HBx–p62–Keap1 complexes immobilize Keap1 in the aggregates thereby prevent Nrf2 from the inhibition by Keap1, leading to the activation of Nrf2.

On the basis of our findings, and in coordination with the observation that HBV results in the accumulation of intracellular p62 by HBx-mediated inhibition of autophagic flux,^19^ we propose a model for HBV-stimulated Nrf2 activation and G6PD expression (Figure 6). Our results suggest a potential impact of HBx on glucose metabolism of hepatocytes, which may be critical to the development of HBV-associated HCC.

## Materials and Methods

### Cell culture, transfection, and RNAi

HepG2, HepG2.2.15, and Huh7 cells were grown in DMEM with 10% FBS at 37 °C under 5% CO_2_. Transient transfection was performed using Lipofectamine 2000 (Invitrogen, Carlsbad, CA, USA) according to the manufacturer's instructions. Cell lines stably expressing HBx-GFP were created by transient transfection followed by selection with 0.8 mg/ml G418 (Sigma-Aldrich, St. Louis, MO, USA).

All the siRNA duplexes were purchased from GenePharma (Shanghai, China). siRNA duplexes designed against conserved targeting sequences were transfected into cells at a final concentration of 20 nM using Lipofectamine 2000 as specified by the manufacturer. The following siRNA duplexes were used: 5′-GCAUUGAAGUUGAUAUCGAU-3′ for p62; 5′-AAGAGUAUGAGCUGGAAAAAC-3′ for Nrf2; and 5′-AAGACCAAUUUCAGCAGACAGTT-3′ for control siRNA. Expression plasmids for shRNAs were made in a pGLVH1/GFP+Puro vector. The targeted sequences were G6PD shRNA 5′-GTCGTCCTCTATGTGGAGAAT-3′ Nrf2 ShRNA 5′-CCGGCATTTCACTAAACACAAC-3′ control shRNA 5′-TTCTCCGAACGTGTCACGTT-3′. Stable shRNA transfectants were selected in medium containing 1 *μ*g/ml puromycin (Sangon Biotech, Shanghai, China).

### Antibodies, reagents, and plasmids

The following antibodies were used: anti-*β*-actin, anti-G6PD (Sigma-Aldrich); anti-HBcAg (Abcam, Cambridge, MA, USA), anti-Keap1 (Cell Signaling, Danvers, MA, USA); anti-Myc, anti-Nrf2 (sc-365949), anti-Lamin B, anti-p62, and anti-Flag (Santa Cruz, Santa Cruz, CA, USA); anti-GFP (BD Biosciences, Franklin Lakes, NJ, USA); goat anti-rabbit IRDye800CW and goat anti-mouse IRDye680 (LI-COR Biosciences, Lincoln, NE, USA). Plasmids with the 1.3mer HBV genomic DNA (pUC19 HBV), the HBx-negative 1.3mer HBV genomic DNA (pUC19 HBVX^−^) and HBx-GFP were described previously.^19, 36^ Dsred-Keap1 was provided by Yue Xiong (University of North Carolina at Chapel Hill, Chapel Hill, NC, USA). For Flag-p62 construction, the p62 coding region was amplified from Cherry-GFP-p62, a gift from Terje Johansen (University of Tromsø, Tromsø, Norway), and was inserted into pCMV-Tag 2A vector using the *Eco*RI and *Xho*I restriction sites. HBx-Myc was made by cloning the cDNA of HBx into pcDNA-3.1 vector using *Eco*RI and *Xho*I restriction sites. The point mutation for Flag-p62 T350A and Dsred-Keap1 N382A was created by PCR-based site-directed mutagenesis using primer: 5′-AAGAAGTGGACCCGTCTGCAGGTGAACTCCAGTCC-3′ (p62-sense), 5′-GGACTGGAGTTCACCTGCAGACGGGTCCACTTCTT-3′ (p62-antisense), 5′-CGTGGGCGGCAGGGCCAACTCGCCCGAC-3′ (Keap1-sense) and 5′-GTCGGGCGAGTTGGCCCTGCCGCCCACG-3′ (Keap1-antisense).

### Human tissues

Frozen fresh liver or liver cancer tissues were anonymously taken from the First Affiliated Hospital, Zhejiang University School of Medicine. Tumor liver tissues and their peripheral non-tumor tissues after surgical resection were collected from HCC patients with chronic HBV infection. Normal liver tissues after surgical resection were collected from patients with liver hemangiomas. The diagnoses were based on clinical laboratory examination. All the human tissues were taken with written informed consent and with the approval of the Medical Ethical Committee of Zhejiang University School of Medicine (No. 1-009). The study was conducted in accordance with the Helsinki Declaration of 1975, as revised in 1983.

### Cell fractionation

About 3 × 10^6^ cells were washed with PBS and resuspended in 200 *μ*l of buffer A (10 mM HEPES pH 7.9, 10 mM KCl, 1.5 mM MgCl_2_, 0.34 M sucrose, 10% glycerol, 1 mM dithiothreitol, 0.1% Triton X-100) supplemented with a complete protease inhibitor cocktail (Roche, Indianapolis, IN, USA). The cells were incubated for 10 min on ice. Nuclei were collected in the pellet by low speed centrifugation (1500 × *g*, 5 min, 4 °C). Nuclei were washed twice with buffer A without 0.1% Triton X-100, then lysed and denatured with sodium dodecyl sulfate (SDS) loading buffer. The Nrf2 levels in nuclei were analyzed by western blotting.

### Western blot and immunoprecipitation

Western blot was performed as described previously.^19^ Briefly, proteins obtained from lysed cells were denatured and loaded on SDS polyacrylamide gel. Afterwards, the proteins were transferred onto PVDF membranes, blocked in TBS-T (150 mmol/L NaCl, 10 mmol/L Tris-HCl pH 7.5, and 0.1% Tween-20) containing 5% (w/v) bovine serum albumin, and incubated with the corresponding primary and secondary antibodies. The specific bands were analyzed by the western blot infrared imaging system (LI-COR Biosciences). For immunoprecipitation, cells were lysed with nonidet P40 lysis buffer (50 mM Tris-HCl pH 7.5, 100 mM NaCl, 1% NP-40, 1 mM EDTA, 1 mM DTT, 10% glycerol) containing protease inhibitors. After centrifugation, the supernatants were incubated with antibody overnight and then Protein A/G agarose for 2 h at 4 °C. Immunocomplexes were washed and analyzed by western blotting.

### G6PD enzyme activity

The enzyme activity of G6PD was assessed using a G6PD assay kit (Sigma, St. Louis, MO, USA) according to the manufacturer's instructions.

### Immunostaining and confocal microscopy

For immunostaining, cells or frozen tissue sections were fixed in 4% formaldehyde. After washing twice with PBS, they were incubated in PBS/FBS (PBS, pH 7.4, containing 10% FBS) to block non-specific sites of antibody adsorption. Then, the cells or sections were incubated with appropriate primary and secondary antibodies in 0.1% saponin as indicated in the figure legends. Images were captured on a Zeiss LSM510 Meta laser scanning confocal microscope (Carl Zeiss, Thornwood, NY, USA) with a 63 Plan Apochromat 1.4 NA objective.

For live-cell imaging, cells in chambers were imaged on a live cell station. Photobleaching was performed using an appropriate laser line at full power. For FRAP analysis, a selected region of a cell was photobleached and the fluorescence recovery of the region was monitored at low intensity illumination. For FLIP analysis, a selected region was repetitively photobleached and the loss of fluorescence from regions outside the photobleached region was monitored. For quantification of fluorescence intensity, non-saturated images were taken with a fully open pinhole, whereas non-quantitative images were obtained with a pinhole diameter equivalent to 1–2.5 Airy units.

### Quantitative PCR

Total cellular RNA was isolated using TRIzol reagent (Invitrogen) and was reverse transcribed using M-MLV Reverse Transcriptase (Promega, Madison, WI, USA) according to the manufacturer's protocol. GAPDH was used as an invariant housekeeping gene internal control. Quantitative PCR was performed using primers as follows: (1) G6PD-sense, 5′-TGACCTGGCCAAGAAGAAGA-3′ and G6PD-antisense, 5′-CAAAGAAGTCCTCCAGCTTG-3′ (2) Nqo1-sense, 5′-GGAGAGTTTGCTTACACTTACGC-3′ and Nqo1-antisense, 5′-AGTGGTGATGGAAAGCACTGCCTTC-3′ (3) Gstm1-sense, 5′-TGCCCATGATACTGGGGTA-3′ and Gstm1-antisense, 5′-GCCACTGGCTTCTG-TCATAAT-3′ (4) Cyp2a5-sense, 5′-ACCAAGGACACCAAGTTTCG-3′ and Cyp2a5-antisense, 5′-AGAGCCCAGCATAGGAAACA-3′ (5) GAPDH-sense, 5′-GGAGCCAAAAGGGTCATCATCT-3′ and GAPDH-antisense, 5′-GAGGAGCCATCCACAGTCTTCT-3′.

### Cell proliferation and colony formation assay

HBx-GFP cell lines were transfected with shRNAs as indicated in the figure legends, and shRNA-expressing cells were selected in medium containing puromycin for 3 weeks. For cell proliferation assay, the cells were seeded in 24-well plates in triplicate at a density of 2000 cells per well in 0.5 ml of medium. Cell number at the indicated time points was determined by counting using a haemocytometer. Colony formation assay in soft agar was performed as previously described.^37^ Briefly, 5 × 10^3^ cells per well were resuspended in DMEM containing 0.3% agar. Then, the suspension was laid over DMEM containing 0.5% agar in each of the triplicate wells of a 6-well plate. The plates were incubated for 14 days in a 5% CO_2_ incubator at 37 °C, with replenishment of medium every 3 days. Colonies were imaged using Nikon ECLIPSE TI microscopy and colonies in three randomly chosen fields per well were counted for quantification.

### Statistical analysis

All data were presented as means±S.E.M. Statistical significance of the differences in the experimental data was determined using the Student's *t*-test. Differences were considered as significant at values of *P*<0.05.

## Abbreviations

HBV: hepatitis B virus
HCC: hepatocellular carcinoma
HBx: hepatitis B virus X protein
G6PD: glucose-6-phosphate dehydrogenase
Nrf2: NF-E2-related factor 2
ROS: reactive oxygen species
Keap1: kelch-like ECH-associated protein 1
PPP: pentose phosphate pathway
HBcAg: hepatitis B core antigen
qRT-PCR: quantitative reverse transcription PCR
FRAP: fluorescence recovery after photobleaching
FLIP: fluorescence loss in photobleaching
Nqo1: NAD(P)H quinone oxidoreductase 1
Gstm1: glutathione S-transferase M1
Cyp2a5: cytochrome P450 2A5
PB1: Phox and Bem1p
UBA: ubiquitin-associated
ZZ: ZZ-type zinc finger
