# Supplementary material for: Hepatitis B virus stimulates G6PD expression through HBx-mediated Nrf2 activation
Source: Cell Death Dis. 2015 Nov 19;6(11):e1980–. doi: 10.1038/cddis.2015.322 (PMC4670929; doi:10.1038/cddis.2015.322)
Supplement: Supplementary Figures Legends [file cddis2015322x1.doc]

**Supplementary Figure legends**

**Figure 1.** HBV/HBx enhances G6PD expression. (**a**) Quantification of *G6PD* mRNA measured by qRT-PCR in normal liver tissues (n = 13), liver tumor (n = 13) or non-tumor tissues (n = 13). Values are mean ± SEM, ***P* < 0.01. (**b** and **c**) Western blot analysis of G6PD expression in HepG2 and HepG2.2.15 cells (b), and L02 cells expressing HBx-Myc, Myc-HBsAg or Flag-HBcAg (c). (**d**) The *G6PD* mRNA level in HBx-expressing L02 cells. The data are presented as mean ± SEM of triplicate experiments. **P < 0.01.

**Figure 2.** HBx stimulates Nrf2 activation. Immunostaining of Nrf2 in L02 cells expressing GFP, HBx-GFP, HBsAg-GFP or HBcAg-GFP. Note the nuclear distribution of Nrf2 in HBx-GFP, but not HBsAg-GFP or HBcAg-GFP cells. Scale bars, 20 µm.

**Figure 3.** Keap1-p62 interaction in HepG2 and HepG2.2.15 cells. (**a**) Coimmunoprecipitation of p62 with Keap1 in HepG2 and HepG2.2.15 cells. (**b**) *Nqo1* mRNA level measured by qRT-PCR in HepG2 and HepG2.2.15 cells with or without p62 RNAi. The data are presented as mean ± SEM of triplicate experiments. **P < 0.01.

**Figure 4.** HBx interacts with p62 through the UBA and PB1 domains of p62. Coimmunoprecipitation of Flag-p62 and Keap1 with HBx-GFP. HBx-GFP-expressing Huh7 cells were transfected with Flag-tagged p62 or each of the truncated p62 mutants. Then the HBx-GFP was immunoprecipited by a GFP antibody and the immunoprecipitates were analyzed by Western blot using anti-Flag and anti-Keap1 antibodies.

**Figure 5.** Colony formation assay. HBx-GFP cell lines expressed indicated shRNAs, and the colony formation of the cells in soft agar was performed. Colonies were imaged using Nikon ECLIPSE TI microscopy.
