# Supplementary material for: Hepatitis B virus stimulates G6PD expression through HBx-mediated Nrf2 activation
Source: Cell Death Dis. 2015 Nov 19;6(11):e1980–. doi: 10.1038/cddis.2015.322 (PMC4670929; doi:10.1038/cddis.2015.322)
Supplement: Supplementary Figures [file cddis2015322x2.pdf]

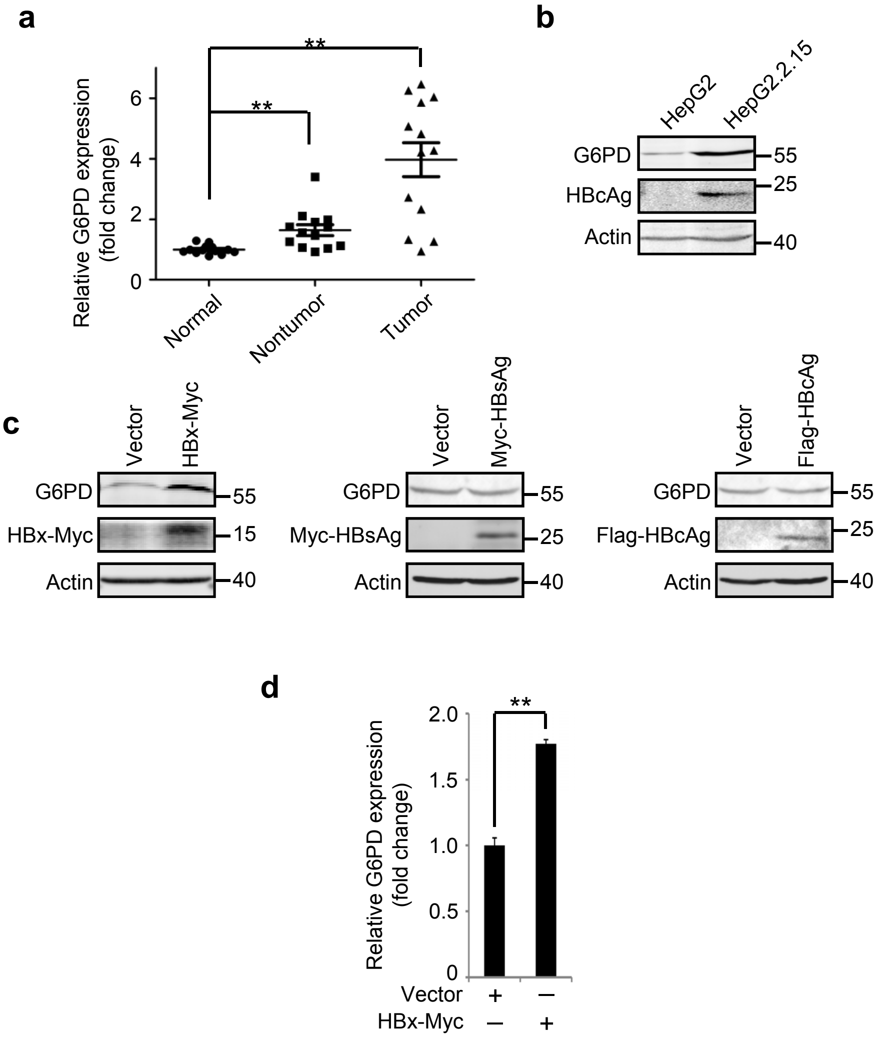

Supplementary Figure 1

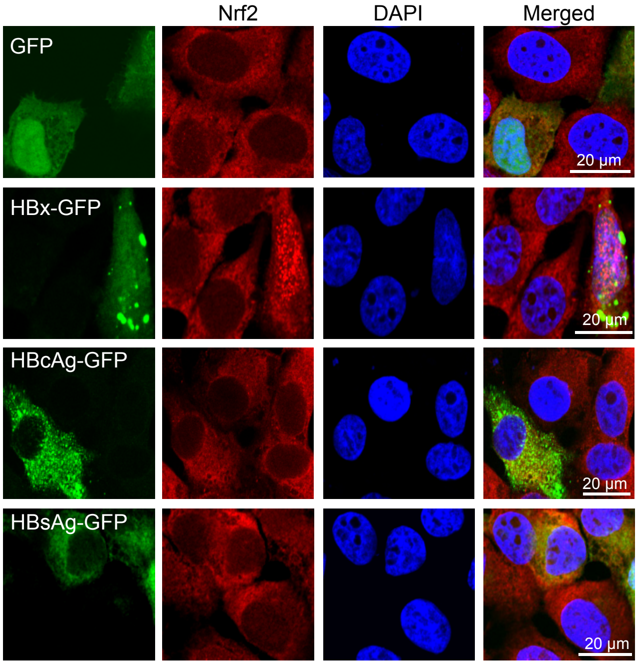

Supplementary Figure 2

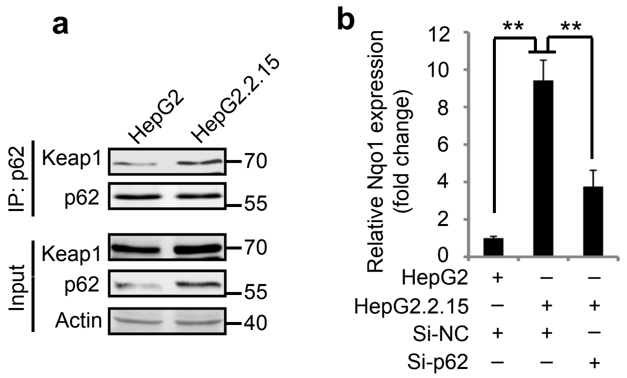

Supplementary Figure 3

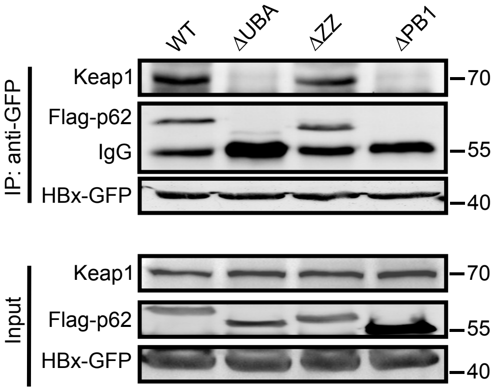

Supplementary Figure 4

Vector, shNC | HBx, shNC | HBx, shNrf2 | HBx, shG6PD

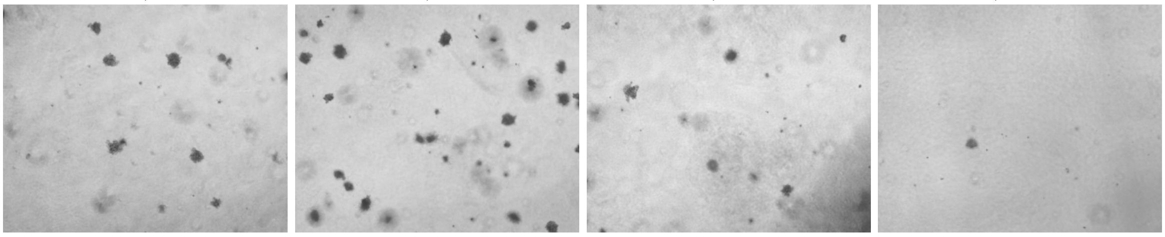

Supplementary Figure 5
